# Immunoreactivity of pan MHC-I epitopes on Crimean-Congo hemorrhagic fever virus glycoprotein C-terminal

**DOI:** 10.3389/fimmu.2026.1693892

**Published:** 2026-03-04

**Authors:** Dongbo Jiang, Yongkai Wang, Haobo Kang, Zhenchi Fang, Jianchang Li, Zilu Ma, Yubo Sun, Yanbo Wang, Zhihe Fu, Yulin Yang, Enqi Guan, Yuanjie Sun, Shuya Yang, Chunmei Zhang, Yusi Zhang, Yun Zhang, Baozeng Sun, Kun Yang, Linfeng Cheng

**Affiliations:** 1 Department of Microbiology, Basic Medicine School, Air-Force Medical University (the Fourth Military Medical University), Xi’an, Shaanxi, China; 2 Department of Immunology, The Key Laboratory of Bio-hazard Damage and Prevention Medicine, Basic Medicine School, Air Force Medical University (the Fourth Military Medical University), Xi’an, Shaanxi, China; 3 Department of Rehabilitation Medicine, Tangdu Hospital, Air-Force Medical University (The Fourth Military Medical University), Xi’an, China; 4 Beijing Institute of Radiation Medicine, Beijing, China; 5 Yingtan Detachment, Jiangxi Corps, Chinese People’s Armed Police, Yingtan, China; 6 Changzheng Hospital, Naval Medical University, Shanghai, China

**Keywords:** Crimean-Congo hemorrhagic fever virus (CCHFV), Gc proteins, immunoreactivity, in silico analysis, MHC-I restriction, pan-MHC-I epitope

## Abstract

**Introduction:**

Crimean-Congo hemorrhagic fever virus (CCHFV) is a tick-borne pathogen causing severe hemorrhagic disease with high mortality. The viral glycoprotein Gc mediates membrane fusion and represents a key target of CD8⁺ T-cell responses. However, systematic identification and comprehensive evaluation of pan-MHC-I-restricted Gc epitopes remain limited.

**Methods:**

An integrated immunoinformatics workflow incorporating five prediction algorithms (IEDB, NetMHCpan4.1, SMMPMBEC, SYFPEITHI, and Rankpep) was applied to identify high-affinity 9-mer epitopes from the CCHFV Gc protein restricted by human HLA-I and murine H2 alleles. Immunogenicity, conservation, toxicity, and allergenicity were assessed using established computational tools. Peptide–MHC interactions were further examined by molecular docking and molecular dynamics simulations. Selected epitopes were experimentally validated by ELISpot assays in BALB/c and SJL mice immunized with a Gc-based DNA vaccine.

**Results:**

Ninety-four human HLA-I and thirty-seven murine H2-restricted dominant epitopes were predicted. Among these, 21 epitopes exhibited high binding affinity, favorable immunogenicity, and broad conservation across viral strains. Most candidates showed low predicted toxicity and allergenicity. Structural analyses supported stable peptide–MHC interactions. ELISpot assays confirmed that several epitopes, including APFILLILF and FVKWKVEYI, elicited significant IL-2 responses, indicating functional T-cell activation.

**Conclusion:**

This study provides a systematic framework for identifying conserved and immunogenic pan-MHC-I-restricted epitopes within the CCHFV Gc protein. The validated candidates may support the rational design of multi-epitope vaccines and contribute to understanding cellular immune responses against CCHFV.

## 1 Introduction

Crimean-Congo hemorrhagic fever virus (CCHFV) is a geographically widespread infectious virus found primarily in Asia, Europe, Africa, and the Middle East (1). CCHFV is the most widespread tick-borne virus (2). Ticks are able to survive in an increasing number of areas due to human activities and geography, leading to the increasing prevalence of CCHFV, which commonly causes Crimean-Congo Hemorrhagic Fever (CCHF), a disease that usually manifests in a staged manner, beginning with influenza-like symptoms, followed by coagulation disorders and, in severe cases, systemic organ failure and even death (3). With a mortality rate of up to 40%, the current status of CCHFV is one of high morbidity and mortality with no clinical means of prevention or treatment (4). In addition, CCHFV is classified as a biosafety level 4 (BSL-4) pathogen, further emphasizing the importance of prevention and research on CCHFV (World Health Organization, n.d.). In addition, the National Institutes of Health (NIH/NIAID) and the World Health Organization (WHO) have listed CCHFV as a priority RNA virus for research (5).

CCHFV is a member of the *Orthonairovirus* genus in the *Nairoviridae* family of the *Bunyavirales* order of viruses with a segmented, negative-strand RNA genome (6). Specifically, the small fragment (S) encodes the nucleoprotein (NP) and the medium fragment (M) encodes the glycoprotein precursor (GPC), which is hydrolyzed during processing and transport to produce Gn, Gc, and other non-structural functional proteins (NSm and GP38, among others). GPC also produces proteins that are considered most valuable for vaccine research. Finally, the large segment (L) encodes the production of the L protein (2). As with other bunyaviruses, Gn and Gc are involved in receptor binding and entry, suggesting that they typically interact with target cells (7). Available data from animal studies indicate that in mice, both Gn and Gc are recognized by immune cells and provide immune protection, but the immune response against Gc leads to prolonged survival in infection models (8). In addition, Gc-associated immune responses can also be found in infected individuals, thereby inducing an effective host immune response against the pathogen. Therefore, Gc is currently considered the most valuable anti-CCHFV immune target to study (9).

MHC class I-restricted CTLs are important effector cells against viral infections. T cell recognition of epitopes and induction of immune responses play key roles in the individual immune system (10). During virus-host symbiosis, viruses interfere with the killing activity of CTLs in different ways, such as modifying or reducing protein sequences or immune epitopes that can be recognized by T cells, thereby evading host clearance (11). These modes of interference compromise the immune homeostasis and immunity of the host. Only high-affinity peptides that can bind to MHC-I molecules have the potential to approach cells and trigger an immune response (12). Therefore, elucidation of high-affinity immunogenic epitopes is essential for the immune response of T cells to CCHFV. At the same time, evolutionary conservation of immunogenic epitopes is a key factor in “herd immunity”, where conserved epitopes provide long-term immune protection to the host during virus-host symbiosis in response to different strains (13).

The key to resistance to CCHFV infection is the activation of cytotoxic T lymphocytes, a process that is influenced by the restriction of MHC-I molecule (14–17). During virus-host interactions, viruses interfere with CTL killing activity in a variety of ways, including altering or reducing protein sequences or immune epitopes recognizable by T cells, thereby evading host clearance (18). Immune evasion by these viruses not only reduces host immunoprotection, but also accelerates viral transmission and increases viral genetic variability. Only high-affinity immune epitopes in conserved regions of the pathogen can be effectively recognized by CTLs and effectively contain the viral immune evasion response (19). Thus, high conservation and high affinity are key factors in epitope evaluation (20). In addition, immunogenicity is the basis for effective antiviral action of an epitope, and highly immunogenic epitopes can be more timely and effective during virus-host interactions, providing stronger protection to the host (11, 21).

From the current case reports, it is clear that CCHF outbreaks usually occur in endemic areas, are transmissible and episodic, but these areas often lack protective measures and research on the CCHFV system (15). Therefore, there is an urgent need to study the relationship between CCHFV and the host and the associated immune protective mechanisms (22). A thorough understanding and knowledge of immune cell interactions with CCHFV is fundamental to respond to and contain viral evolution and regional epidemic (7, 23). The use of bioinformatics allows the systematic characterization of CCHFV-related properties with the aim of informing the subsequent provision of immunoprotection by elucidating the localization of potential epitopes, their selection and the assessment of immune potential (24). In recent CCHFV vaccine development, epitope vaccine design based on bioinformatics-based computational design tools has become a future trend (20).The advantages of epitope design are not limited to the ability to improve precise localization and efficient induction, as well as broad spectrum and higher safety, making it a safe, reliable and efficient means of responding to CCHFV (21, 25).

In this study, based on bioinformatics approach and animal experiments, we screened the valuable CCHFV standard strain IbAr10200 Gc 9-mer epitopes. The affinity, immunogenicity, and conservation of the Gc 9-mer epitopes were preliminarily analyzed using various informative immunological tools. The eligible epitopes were thoroughly and systematically evaluated, and those deemed valuable for the study were selected based on immunological assessments from multiple perspectives and detailed epitope-by-epitope performance analyses. The cellular immune responses induced by epitopes were verified by enzyme-linked immunospot (ELISpot) assay to refine the epitope screening system. The epitope screening evaluation system and the selected epitopes provide insights for the subsequent in-depth study of virus and the design of epitope vaccines.

## 2 Material and methods

### 2.1 Amino acid sequence retrieval of CCHFV Gc

The CCHFV glycoprotein IbAr10200 (Gc, accession number: AF467768.2) was retrieved from the NCBI nucleotide database and used as input for the sequential bioinformatics tools for epitope prediction, conservation analysis, and experiments. To calculate the conservation rank of predicted epitopes overlapping Gcs, protein sequences of all CCHFV isolates were obtained from NCBI.

### 2.2 Screening for high affinity CCHFV Gc 9-mer Epitopes

The intrinsic nature of the epitope (affinity for MHC) and the intrinsic nature of the host (frequency of naive precursors) together determine the immunodominance of the CD8+ T cell response (26). Thus, high-affinity epitopes are more likely to activate T cells to generate an immune response. We first segmented the Gc-generating epitopes separately using five different algorithms, then performed affinity calculations of MHC-I molecules to the epitopes, and then categorized the statistics in terms of different MHC-I molecules (including 9 human HLA-I superfamilies and 6 mouse H-2 genotypes). Bioinformatics tools for affinity screening were included in authoritative databases IEDB(IEDB.org), SMMPMBEC from the IEDB(immuneepitope.org), NetMHCpan4.1(NetMHCpan 4.1 - DTU Health Tech - Bioinformatic Services), SYFPEITHI(SYFPEITHI: Epitope prediction) and Rankpep(Rankpep (ucm.es)). To ensure transparency and reproducibility, the specific binding affinity thresholds (%Rank or affinity scores) used to define the “top 2%” criterion for each prediction algorithm are provided in **Supplementary Table 1**. This table details the exact cutoff values (e.g., %Rank ≤ 2 for IEDB/SMMPMBEC/NetMHCpan4.1/Rankpep; the top 2% of high-affinity epitopes for SYFPEITHI) derived from the respective algorithm’s distribution-based ranking.

### 2.3 Immunogenicity analysis

Immunogenicity (T cell recognition) is an important factor in determining whether pMHCs can be targeted in immune responses (12). Even peptides with high affinity may not adequately induce an immune response (27). Therefore, peptides with high immunogenicity are important for activating T cell responses when screening epitopes (28). Immunogenicity is determined by its chemical and host factors. Small peptides are mainly determined by amino acid sequence (29). In this study, immunogenicity analysis used two analysis software, PAAQD2.0(GitHub - masato-ogishi/PAAQD2: A code-refined version of PAAQD) and IEDB(Class I Immunogenicity (iedb.org), to calculate the immunogenicity of 9-mer peptide epitopes. The screened peptides were considered immunogenic with a probability score of >0.5 as a positive criterion, and intersected in the two databases; Otherwise, they are not considered immunogenic.

### 2.4 Conservation Analysis

We used BLASTp (Protein BLAST: search protein databases using a protein query) to analyze the evolutionary conservation of the dominant epitopes screened at the initial stage. The evaluation criterion for inter-specific conservation was Orthonairovirus (taxid: 1980517), excluding CCHF orthonairovirus (taxid: 1980519). For intraspecific conservation, the criterion was CCHF orthonairovirus (taxid: 1980519), excluding CCHFV strain IbAr10200 (taxid: 652961). The conserved E-value threshold of <10^-5^ was adopted as standard in immunoinformatics (30) to ensure statistically significant sequence homology, as sequences with E-value >10^-5^ are typically considered non-significant in homology-based conservation studies. The screened epitopes were categorized according to the judgment of interspecific and intraspecific conservation into four categories: interspecific and intraspecific conservative, neither interspecific nor intraspecific conservative, interspecific conservative intraspecific non-conservative, and interspecific non-conservative intraspecific conservative. In the resulting tables, ‘+’ represents conservation and ‘-’ represents non- conservation.

### 2.5 Clustering and grouping studies of CCHFV Gc peptides and Pan-MHC molecules

The complexity and polymorphism of MHC molecules result in differences in affinity for different epitopes. For intuitive understanding and further grouping studies, we used the big data mapping engine TBtools to perform bidirectional hierarchical clustering analysis of the binding affinity of MHC-I superfamily and CCHFV Gc-related peptides (31). Using statistical analysis, we processed the affinity ranking data with base 2 logarithmic and Z-score minus transformation. Subsequently, after statistically processing the affinity scores, we performed a two-way hierarchical cluster analysis of the 9-mer peptides of CCHFV based on *Euclidean distance*. The results of the analysis showed that the closer the scores were to high values on the graph, the stronger the affinity between the 9-mer peptides and the MHC-I molecules. We analyzed 33 pan-MHC-I molecules interacting with 636 CCHFV Gc epitopes and visualized the results of the analysis and groupings using heatmaps.

### 2.6 Toxicity and sensitization of peptides

Peptides have proven to be one of the most promising tools for the treatment and prevention of various diseases. However, toxicity remains a major obstacle to the development of peptide-based therapeutics and vaccines (32).At the same time, allergic disease involves a complex interplay of intrinsic and extrinsic factors that contribute to disease onset and symptom triggering (33), so the exclusion of allergenic epitopes is an important requirement when screening vaccine candidates.To ensure the safety of the pre-screened dominant Gc epitopes, we evaluated the toxicity and allergenicity of all high-affinity, strongly immunogenic and evolutionarily conserved 9-mer peptides using the web-based tools ToxinPred 3.0(ToxinPred3.0)and AlgPred 2.0(AlgPred2(iiitd.edu.in)), respectively. All 21 epitopes were submitted to ToxinPred 3.0 in FASTA format using the peptide toxicity prediction module with default deep learning–based settings, and peptides predicted as toxic by the server were considered to have potential toxicity. In parallel, allergenicity was predicted using the hybrid (Random Forest + BLAST + MERCI) model implemented in AlgPred 2.0, applying the default parameters recommended by the server; peptides classified as allergenic were considered sensitizing. All predicted toxic and/or allergenic epitopes were recorded and tabulated for subsequent statistical and comparative analysis.

### 2.7 Modeling and evaluation of pMHC-I molecular docking

Molecular docking simulations were performed to model the binding conformations of dominant epitopes within the peptide-binding groove of representative MHC-I allotypes using HPEPDOCK 2.0 (34, 35). Given the high-throughput nature of our screening pipeline, we prioritized computational efficiency while maintaining prediction accuracy through a two-tiered validation strategy:(i) initial blind docking of 9-mer dominant epitope sequences against archived MHC-I structures from the PDB library, followed by (ii) energy minimization and consensus scoring to refine top poses. The 100 highest-scoring models per epitope-allele pair were generated, with the top 10 ranked by HPEPDOCK’s composite score selected as high-confidence predictions. This allowed us to establish a predictive modeling framework and define a binding-affinity-based threshold for pan-specific MHC-I epitope interactions.

We use archived data from the protein library with MHC-I allele for analog docking. The 9-mer dominant epitope sequence and allele interaction and stratification algorithm were used to carry out blind protein-peptide docking by entering the 9-mer dominant epitope sequence in HPEPDOCK2.0 (HPEPDOCK 2.0 (hust.edu.cn)), and the docking simulation modeling and threshold were obtained for analysis and screening (36). The resulting modeling and threshold docking tests yielded 100 simulation models, and the first 10 were selected as high-confidence docking results.

For well-docked pMHC complexes, we subsequently conducted classical molecular dynamics simulations to assess the stability of docking-derived binding modes. For each trajectory, we calculated the root mean square deviation (RMSD) of the binding peptide chain’s backbone relative to the initial docking conformation during the simulated equilibrium phase. For each complex, we reported the average peptide RMSD, RMSD standard deviation, and the proportion of frames within the steady-state region as quantitative descriptors of structural stability.

### 2.8 Multi-sequence alignment of CCHFV spike glycoprotein

To verify the evolutionary conservation of the Gc site in CCHFV IbAr10200 strain against 99 independent isolates retrieved via BLASTp search in NCBI, we employed ClustalX2.1 (Clustal W and Clustal X Multiple Sequence Alignment) for multiple sequence alignment. The analysis focused on identifying sequence differences between hotspot variants and globally prevalent strains. In addition, we analyzed the details of the comparisons using WebLogo (WebLogo - Create Sequence Logos (berkeley.edu)). The features obtained from the output highly reflect the frequency of amino acid mutants occurring in the CCHFV variant strains. Based on our amino acid comparison results, we further analyzed the alteration of HLA-I molecular affinity by highly mutated sites. The binding affinity δ between strain IbAr10200 and the corresponding HLA-I and 9-mer epitope variants is illustrated using a heatmap generated by TBtools, an algorithm that calculates binding affinity on a logarithmic basis 2. Negative δ values of %Rank indicate that the epitopes of strain IbAr10200 have better binding affinity, while positive values indicate that mutated amino acids have better binding affinity for the corresponding MHC-I molecules with better binding affinity. Finally, to determine whether the amino acid changes altered the binding advantage of the original epitopes, we analyzed and counted all 9 peptides that could be affected by the mutated sites.

### 2.9 CCHFV DNA vaccine designed and prepared

A DNA vaccine for pVAX-Gc_CCHFV_ was synthesized using the pVAX1 vector with the gene for CCHFV Gc inserted (GenBank number: AF467768.2). The vaccine, which was synthesized more than once, was verified to be free of mutation sites by gene sequencing. The prepared plasmids were isolated and purified using the Plasmid Maxi Kit (DP117; TIANGEN, Beijing, China), stored at low temperature until use, and tested for concentration and purity.

### 2.10 Selection of animal models and immunization schedule

Healthy female mice (BALB/c and SJL; 6–8 weeks old) were obtained from the Laboratory Animal Center of the Fourth Military Medical University (Xi’an, China). Both experimental and control groups were equipped with 6 mice, which were vaccinated with pVAX-Gc_CCHFV_ vaccine at 0, 3 and 6 weeks, respectively. Blood and organ taken of mice were performed at 8 weeks. The cellular immune response after immunization was assessed by ELISpot assays.

### 2.11 Peptides and ELISpot assay

HLA- and H2-restricted dominant epitopes (ChinaPeptides, China) of CCHFV Gc were artificially synthesized according to the screened dominant epitopes. ELISpot detects antibody-secreting cells (ASCs) and cytokine-secreting cells (CKs) at the single-cell level, diluted in a sterile bench with synthetic dominant epitopes diluted with 20 μg/mL phosphate-buffered saline (PBS) and interferon IL-2 ELISpot reagent from BD Pharmingen (Franklin Lakes, NJ, USA). Specifically, 5 μg/mL (1:250 dilution) IL-2 specific capture antibody was added to the Millipore 96-well PVDF ELIspot plate at 4 °C overnight. After immunization, the spleen was harvested, the spleen was crushed, the red blood cells were lysed and washed, the spleen cells were centrifuged, and the spleen cells were resuspended. For the negative control, 100 μL of the polypeptide from step 7 was added as pure RPMI-1640 at 100 μL/well per well. The positive control was Canavalin A (10 μg/mL), then plate the cells evenly, 100 μL per well, 1*10^5^ ~ 2*10^6^ cells/mL, tap the edge of the plate to spread the cells evenly, and then place in the incubator, 37 °C, 5% CO2 culture for 24 hours. After incubation, biotin-labeled detection antibody is diluted with diluent to 2 μg/mL (original concentration 0.5 mg/mL, 1:250 dilution), and then 100 μL per well is added and incubated for 2 hours at room temperature. After washing with PBST, incubate with streptavidin-horseradish peroxidase (1:100 dilution) for 1 hour, add substrate [3-amino-9-ethylcarbazole (AEC); BD - Pharmingen], wash with water to stop the reaction. After completion of the reaction, IL-2 spots are counted using the ELISpot Reader Classic-ELR06 (AID, Strasbourg, Germany). The results will indicate responsive cells. Finally, the data were analyzed and plotted using Prism 8.0.1 (GraphPad, San Diego, CA, USA) and Student’s t-test based on three replicates.

ELISpot assays comprised three biological replicates per group (n=3 mice/group). Each biological replicate under each stimulation condition within a group underwent technical triplicate testing. Complete RPMI-1640 medium served as the negative (medium) control, concanavalin A (ConA, 10 μg/mL) as the positive control, and a non-epitope 9-peptide (20 μg/mL) as the irrelevant peptide control to assess non-specific peptide reactivity. The SFU count from the irrelevant peptide control served as a critical threshold to exclude non-specific responses. A positive specific T-cell response was determined when the SFU count in the experimental group exceeded this threshold and inter-group differences were statistically significant.

### 2.12 Animal ethics statement

All animal experiments follow the “3R” (Reduction, Replacement, Refinement) principle. All animals are guaranteed fresh drinking water and food, comfortable and sterile habitat, access to prevention and rapid treatment, adequate space, appropriate facilities and social partners of the same kind, and no mental depression and distress in the animals. The animals and procedures used in the experiments were approved by the Animal Ethics Committee of the Fourth Military Medical University and followed internationally recognized guidelines and government animal ethics regulations.

## 3 Results

### 3.1 Prediction of the epitopes for mouse H2 and alleles of major HLA-I supertypes

In epitope evaluation, high-affinity epitopes are one of the characteristics that determine immunoreactivity, and our study analysed the binding between CCHFV Gc 9-mer peptide and MHC as the basis for T cell epitope prediction. Through the five integrated bioinformatics platforms in the methodology, the top 2% of high-affinity epitopes outputted by each platform were selected, and after counting, epitopes with three or more duplicates on the five platforms were selected and considered as dominant epitopes. **Tables 1** and **2** show the number of dominant epitopes identified by the prediction tool. Statistical and computational analyses showed that 94 dominant epitopes were identified on human HLA-I haplotypes and 37 dominant epitopes were identified on mouse H2 haplotypes. In addition, HLA-A3 and HLA-B44 had the highest number of dominant epitopes, 21 and 25, respectively. Among the H2 haplotypes, H2-Kd and H2-Kk had the highest number of dominant epitopes, 8 and 10, respectively.

**Table 1:** Numbers of murine MHC-I dominant epitopes of CCHFV Gc.

MHC-I Haplotypes	Prediction tools	Candidate epitopes	Dominant epitopes
H-2-Db	IEDB	6	6
	NetMHCpan	6	
	Rankpep	3	
	SMMPMBEC	6	
	SYFPEITHI	6	
H-2-Dd	IEDB	5	5
	NetMHCpan	5	
	Rankpep	3	
	SMMPMBEC	3	
	SYFPEITHI	/	
H-2-Kb	IEDB	7	7
	NetMHCpan	7	
	Rankpep	4	
	SMMPMBEC	6	
	SYFPEITHI	/	
H-2-Kd	IEDB	8	8
	NetMHCpan	8	
	Rankpep	6	
	SMMPMBEC	5	
	SYFPEITHI	8	
H-2-Kk	IEDB	10	10
	NetMHCpan	10	
	Rankpep	3	
	SMMPMBEC	7	
	SYFPEITHI	7	
H-2-Ld	IEDB	5	5
	NetMHCpan	5	
	Rankpep	1	
	SMMPMBEC	1	
	SYFPEITHI	5	

**Table 2:** Numbers of HLA-I dominant epitopes of CCHFV Gc.

MHC-I Haplotypes	Prediction tools	Candidate epitopes	Dominant epitopes
HLA-A1	IEDB	36	16
	NetMHCpan	36	
	Rankpep	12	
	SMMPMBEC	36	
	SYFPEITHI	12	
HLA-A2	IEDB	36	16
	NetMHCpan	48	
	Rankpep	48	
	SMMPMBEC	36	
	SYFPEITHI	12	
HLA-A3	IEDB	60	21
	NetMHCpan	72	
	Rankpep	60	
	SMMPMBEC	60	
	SYFPEITHI	36	
HLA-A24	IEDB	36	11
	NetMHCpan	36	
	Rankpep	24	
	SMMPMBEC	36	
	SYFPEITHI	12	
HLA-B7	IEDB	48	23
	NetMHCpan	48	
	Rankpep	48	
	SMMPMBEC	48	
	SYFPEITHI	48	
HLA-B8	IEDB	12	6
	NetMHCpan	12	
	Rankpep	12	
	SMMPMBEC	0	
	SYFPEITHI	0	
HLA-B15	IEDB	24	13
	NetMHCpan	24	
	Rankpep	24	
	SMMPMBEC	12	
	SYFPEITHI	12	
HLA-B44	IEDB	36	25
	NetMHCpan	36	
	Rankpep	36	
	SMMPMBEC	24	
	SYFPEITHI	12	
HLA-B58	IEDB	24	14
	NetMHCpan	24	
	Rankpep	24	
	SMMPMBEC	24	
	SYFPEITHI	12	

### 3.2 Immunogenicity analysis of the CCHFV 9-mer Gc epitope

Once a dominant epitope is processed and presented by antigen-presenting cells during immunization, strong immunogenicity means that it can be recognized by the receptor and elicit an effective specific immune response. Therefore, immunogenicity testing was performed on high-affinity human and mouse restricted epitopes. Fifty-four out of 94 human high-affinity epitopes and 23 out of 37 mouse epitopes were considered to be highly immunogenic (**Supplementary Table 2**).

### 3.3 Analysis of the evolutionary conservation of CCHFV Gc 9-mer antigen

To ensure that the screened dominant epitopes remained unchanged during viral evolution, we performed inter-& intra-species conservation judgments for CCHFV on the predicted high-affinity 9-mer peptides screened by the BLASTp conservation prediction algorithm according to the criteria described in Methods. The statistical results are shown in **Table 3** and the epitopes listed are the results of the epitope conservation analysis using default settings. The dominant epitopes of the screened human and mouse viruses were classified into four categories (Neither interspecific nor intraspecific conservative, interspecific intraspecific conservative, interspecific conservative intraspecific unconservative, intraspecific conservative intermediate unconservative) according to whether they were conserved interspecies or not and whether they were conserved intraspecies or not. Statistically, the HLA-restricted high-affinity 9-mer peptides were much more conserved than the H-2, especially with the highest number of HLA-B7 (7), while only interspecies and intraspecies conserved dominant epitopes were detected in H2-Kd, H2-Kk and H2-Ld.

**Table 3:** Numbers of HLA-I dominant epitopes of CCHFV Gc.

MHC-I	interspecies- intraspecific-	interspecies- intraspecific+	interspecies+ intraspecific-	interspecies+ intraspecific+
H2-Db	1	1	0	0
H2-Dd	0	1	0	0
H2-Kb	1	1	0	0
H2-Kd	1	1	0	2
H2-Kk	1	2	0	2
H2-Ld	1	0	0	1
HLA-A1	0	2	0	4
HLA-A2	2	2	0	4
HLA-A3	2	2	0	3
HLA-A24	1	4	0	3
HLA-B7	3	3	1	7
HLA-B8	1	0	0	1
HLA-B15	0	3	0	2
HLA-B44	2	2	0	4
HLA-B58	1	1	0	2

### 3.4 Hierarchical clusters reveal interactions between MHC-I molecules and individual CCHFV 9-mer peptide antigens.

**Figure 1:**
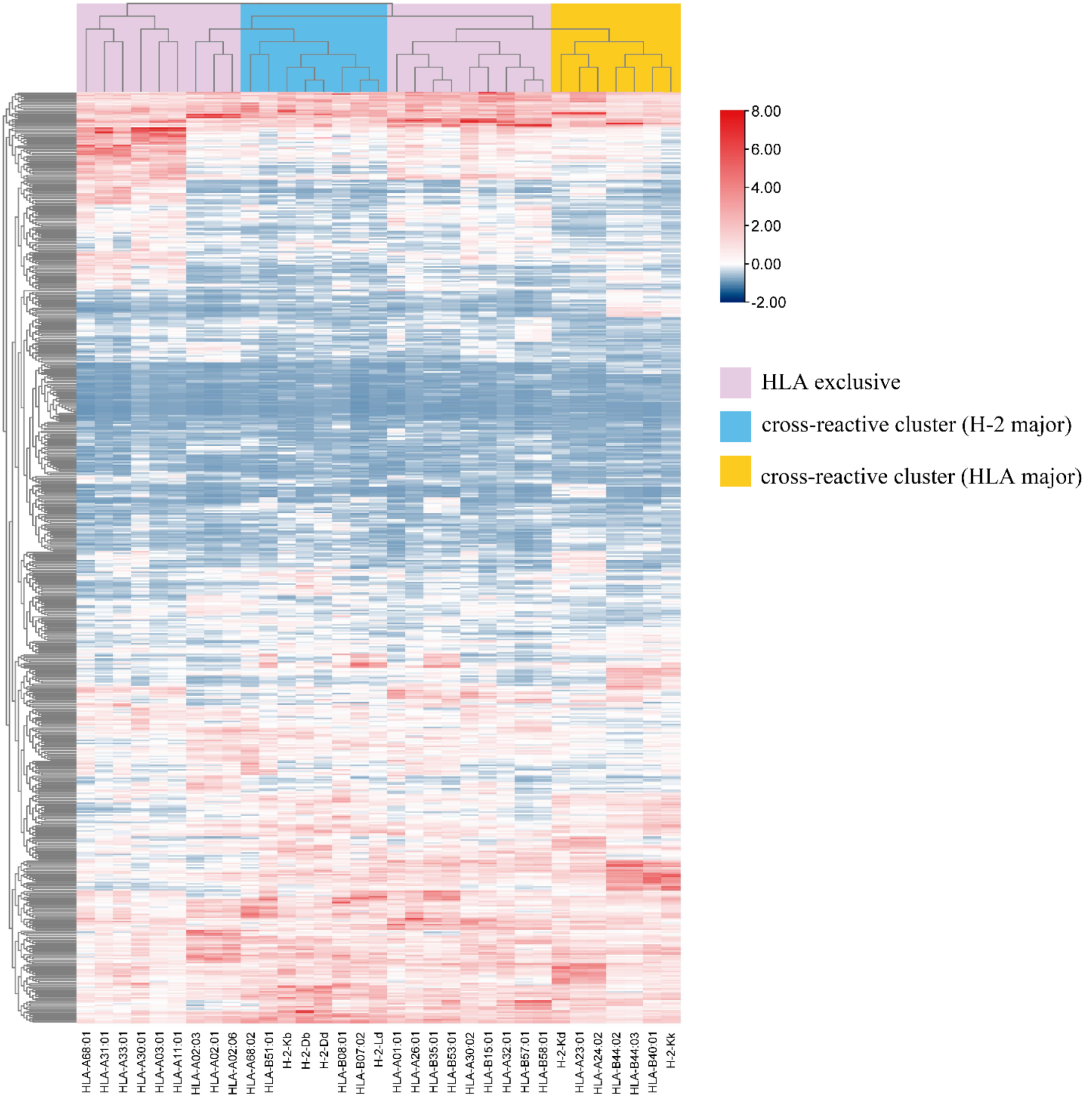
Interaction between CCHFV Gc 9-mer peptides and pan-MHC-I supertypes. Thirty-three MHC-I subtypes were assigned to three clusters, including HLA-I-exclusive and two cross-reactive clusters (HLA major and H2 major). This demonstrates the similarities and differential characteristics among various MHC molecules in their capacity to present antigens. Red represents strong affinity, blue represents weak affinity.

To investigate the patterns and changes of dominant epitopes in pan-MHC I supertype treatments, the present study demonstrated the binding capacity of different MHC-I molecules to the dominant epitopes. Hierarchical cluster analysis was performed to explore similarities and differences during the process of ligating the epitopes to the MHC molecules. As shown in **Figure 1**, Thirty-three MHC-I subtypes were assigned to three clusters, including HLA-I-exclusive and two cross-reactive clusters (HLA major and H2 major). In the HLA-I-exclusive cluster, the HLA-A3 (-A6801, -A3101, -A3301, -A3001) scores were similar to those of HLA-A2 (-A0201, -A0202, -A0206, and-A6801), more so than those of other HLA-A1 superfamilies, suggesting an HLA-A3 like characteristic in CCHFV Gc processing. HLA-B7 (-B3501, -B5301), HLA-A1 (-A2601, -A0101) had similar antigen presentation results; HLA-A1 (-A3002), HLA-B15 (-B1501), HLA-A3201 and HLA-B58 (-B5701, -B5801) had similar antigen presentation results. As for the cross-reactive clusters (H-2 major), H2-Ld scored similar to HLA-B7 (-B0702) and HLA-B8 (-B0801); H2-Db, H2-Dd and H2-Kb went together as H2 exclusive manner. However, in cross-reactive clusters (HLA major), H2-Kk scored similar to HLA-B44 (-B4001, -B4402, -B4403). These similar trends in clustered performances suggest that these superfamilies are represented in both human and mouse cross-reactivity. The fact that antigens presenting the same CCHFV Gc elicit closer cellular immune responses has important implications for animal studies and vaccine design.

### 3.5 Predicting dominant epitope toxicity and lethality

We initially prioritised 21 high-affinity, intra- and inter-species conserved Gc-derived MHC-I 9-mer epitopes. To ensure safety for downstream vaccine design, toxicity and allergenicity were evaluated (**Table 4**) using ToxinPred 3.0 (deep-learning peptide toxicity) and AlgPred 2.0 (hybrid RF/BLAST/MERCI) on the pre-screened set. ToxinPred 3.0 classified six epitopes as potentially toxic—WPHSRNWRC, RNWRCNPTW, TEAIVCVEL, MGDWPSCTY, SLCFYIVER, and RRTRGLFKY—while the remainder exhibited low toxicity. AlgPred 2.0 predicted allergenic potential only for two epitopes, VEYIKTEAI and QPQSILIEH, with the remaining 19 identified as non-allergenic, thereby underscoring the overall low sensitization risk of the selected epitope panel. Notably, LEERTGISW, MYSPVFEYL and MLSGIFGNV exhibited exceptionally low toxicity and allergenicity scores, making them particularly safe dominant epitopes for inclusion in subsequent multi-epitope vaccine designs, while those flagged for toxicity and/or allergenicity were deprioritized.

**Table 4:** Comprehensive assessment table for toxicity and allergenicity of dominant epitopes.

Epitopes	Toxicity Score	Character	Allergenicity Score	Character
TSLSIEAPW	0	Non-Toxin	0.31	Non-Allergen
LEERTGISW	0.295	Non-Toxin	0.3	Non-Allergen
MYSPVFEYL	0.175	Non-Toxin	0.27	Non-Allergen
LHKEWPHSR	0.325	Non-Toxin	0.27	Non-Allergen
WPHSRNWRC	0.7	Toxin	0.28	Non-Allergen
RNWRCNPTW	0.735	Toxin	0.37	Non-Allergen
DVKDLFTDY	0.3	Non-Toxin	0.38	Non-Allergen
FVKWKVEYI	0.375	Non-Toxin	0.3	Non-Allergen
VEYIKTEAI	0.275	Non-Toxin	0.41	Allergen
TEAIVCVEL	0.46	Toxin	0.34	Non-Allergen
RFNLGPVTI	0.26	Non-Toxin	0.4	Non-Allergen
IEEGFFDLM	0.305	Non-Toxin	0.34	Non-Allergen
EPHFNTSWM	0.23	Non-Toxin	0.32	Non-Allergen
MGDWPSCTY	0.59	Toxin	0.4	Non-Allergen
EPDELTVHV	0.175	Non-Toxin	0.4	Non-Allergen
SLCFYIVER	0.54	Toxin	0.28	Non-Allergen
QPQSILIEH	0	Non-Toxin	0.43	Allergen
ILIEHKGTI	0	Non-Toxin	0.33	Non-Allergen
MLSGIFGNV	0	Non-Toxin	0.16	Non-Allergen
APFILLILF	0.005	Non-Toxin	0.35	Non-Allergen
RRTRGLFKY	0.405	Toxin	0.34	Non-Allergen

### 3.6 Molecular Docking of Dominant Epitope Peptides to the MHC

**Figure 2:**
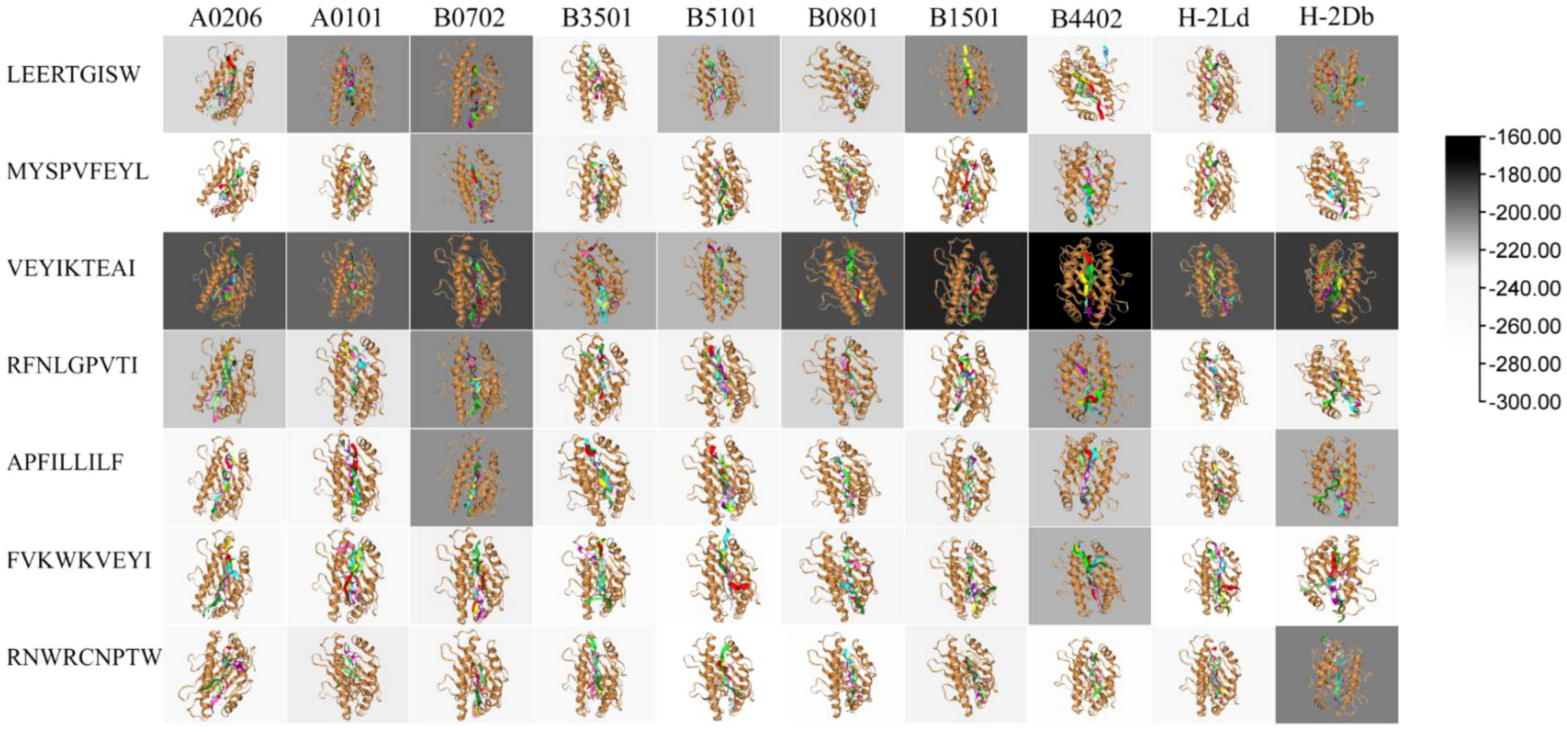
Schematic representation of the molecular interaction between epitopes and MHC molecules, the background showing heatmap of the maximum binding free energy of molecular docking. Through computational docking simulations, the top ten thermodynamically favorable binding poses were identified, wherein the maximum binding free energy of binding reflects the optimal ligand-receptor interaction energetics.

We performed molecular docking simulations and functional scoring using HPEPDOCK 2.0 in Methods to estimate the binding affinity between the dominant epitope and the 3D structure of MHC -I molecules. Following molecular docking, the binding conformations of the 3D structure and the dominant epitope were employed to generate a binding model, which depicted the cluster of binding sites on MHC-I molecules. Following a comprehensive evaluation, we selected the nine epitopes that exhibit high affinity, strong immunogenicity, and conservation across both inter- and intra-species variants, representing the most representative candidates among the 21 identified epitopes. The molecular simulations of the docking and binding site clusters are presented in **Figure 2**. Each docking model provided the top 10 epitopes of MHC-I molecules and the corresponding binding models. The major docking epitopes include LEERTGISW, MYSPVFEYL, VEYIKTEAI, RFNLGPVTI, APFILLILF, FVKWKVEYI, and RNWRCNPTW.

The visual 3D model of molecular docking does not provide a comprehensive understanding of the strength of the binding affinity of the same MHC to different dominant epitopes. Therefore, we performed a cluster analysis of the state with the lowest molecular binding energy (best docked) to represent the docking results in a more visually intuitive manner (background in **Figure 2**). A comparison of the epitopes revealed that the epitopes RNWRCNPTW and FVKWKVEYI exhibited superior ligand-receptor affinity under the algorithm in their respective binding poses with MHC-I molecules. In terms of comparison between MHC-I molecules, the best binding poses for the three alleles HLA-B*0702, HLA-B*4402, and H2-Db exhibited weaker binding affinities than the other groups for the majority of the epitopes. Furthermore, the binding affinities for the other groups were found to be superior to these three groups of alleles under this algorithm.

To directly correlate docking scores with dynamic stability, we combined docking analysis with the RMSD metric derived from molecular dynamics simulations for eight representative pMHC complexes. **Supplementary Figure 1** (box plots of steady-state peptide RMSD distributions across these eight pMHC complexes) shows that mean peptide RMSDs remained within the 1.25–3.35 Å range with low standard deviations, indicating that high-affinity epitopes (e.g., FVKWKVEYI, RNWRCNPTW, and VEYIKTEAI) consistently maintained low to moderate RMSD values throughout simulations and remained stably anchored within the MHC-I groove. **Supplementary Figures 2**–**10** present comprehensive molecular dynamics analyses for each of the eight pMHC complexes, including backbone RMSD over time, backbone RMSF profiles, principal component analysis (PCA) of backbone conformational space, and radius of gyration (Rg) trajectories. These condensed plots and representative structural snapshots provide detailed insights into the dynamic behavior of each complex, confirming that high-affinity epitopes maintain stable backbone conformations and consistent compactness throughout the simulations, thereby reinforcing the link between predicted binding affinity and structural stability. These visualizations further highlight the correspondence between structural stability and docking/binding affinity.

### 3.7 Differences in affinity between standard CCHFV IbAr10200 and mutant strains

**Figure 3:**
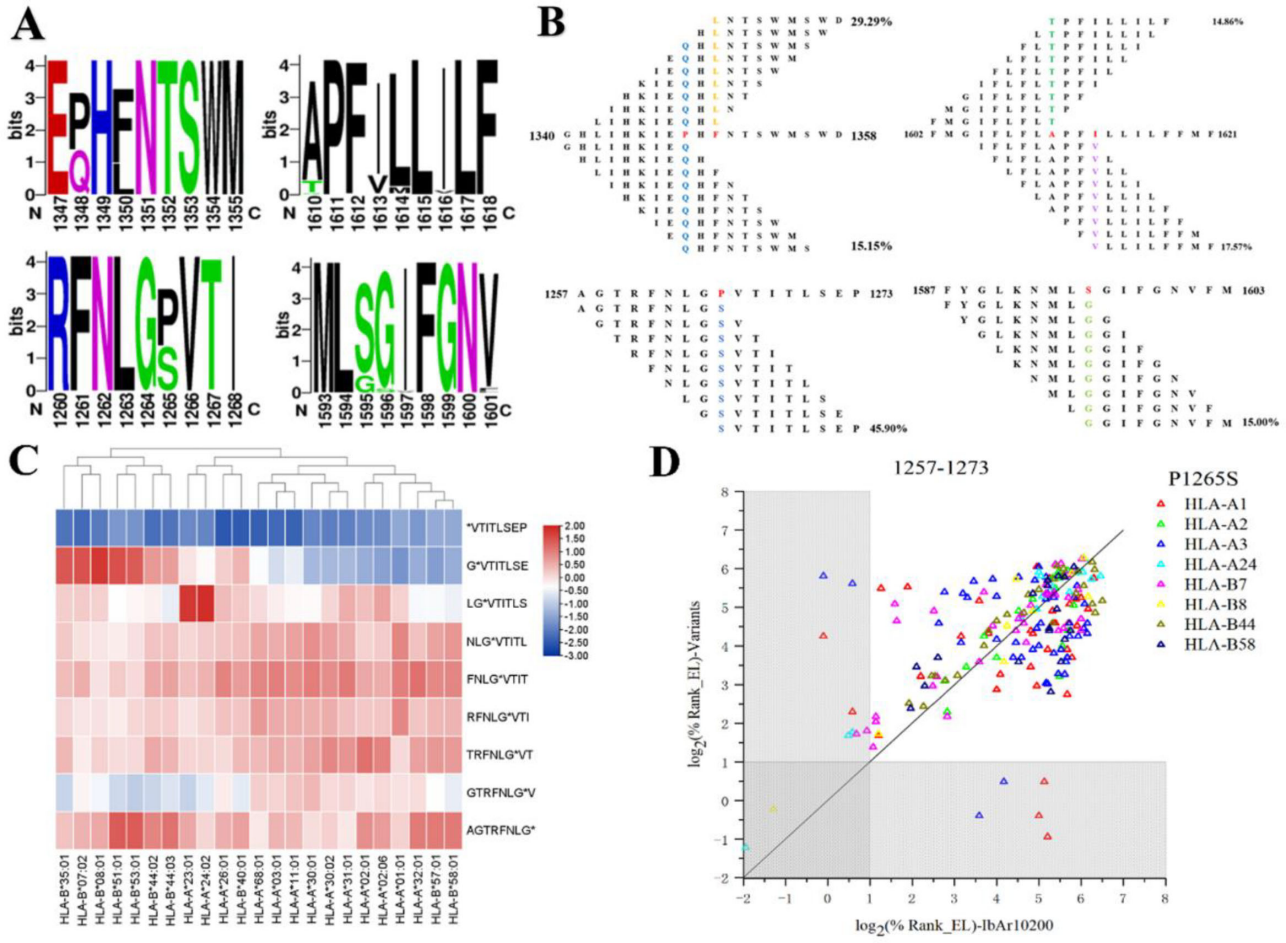
Variant analysis of the dominant epitope in CCHFV. **(A)** WebLogo diagram of variant analysis of dominant epitope peptides. **(B)** Mutation frequency and range of effect of high frequency mutation sites. **(C)** Clustering of MHC-I affinity differences before and after the aa1257-aa1265 mutation. (Blue indicates mutations resulting in decreased affinity, red indicates mutations resulting in increased affinity. **(D)** Scatter plot of MHC-I affinity differences before and after the aa1257-aa1265 mutation. (Each data point in the scatterplot corresponds to an analyzed epitope, and points within the gray area indicate that the epitope has high affinity (within the top 2% of affinity). The closer the data points are to the y = x line, the closer the IbAr10200 strain is to the mutant strain in terms of MHC-I binding ability.).

The amino acid sequence of CCHFV IbAr10200 strain was compared with that of 99 other mutant strains. The comparison results of 21 selected dominant epitopes were observed, and four dominant epitopes with mutation sites were selected (**Figure 3**), and the high-frequency mutation sites were counted and analyzed. Several high-frequency mutation sites were found, such as P1265S, P1348Q and F1350L, and the mutation effect areas and mutation rates of these sites were counted (**Figure 3**), and it was found that the largest mutation effect areas were aa1340-aa1359, with more than half of the mutation rates. This was followed by the highly mutated locus P1265S with a mutation rate of 45.098%.

By comparing the effect of the IbAr10200 strain and the mutant strain with the highly mutated locus P1265S with HLA on epitope affinity, it was found that the mutation resulted in an alteration of the affinity for HLA (**Figure 3C** heat map and **Figure 3D** scatter plot). Among the aa1257-aa1273 peptides, the heatmap showed that PVTITLSEP had the least altered affinity for HLA I. The top 2% of HLA-A1 in the scatterplot had the highest affinity, and it is possible that their affinity was less affected before and after the mutation. Among the amino acid sequences associated with the aa1257-aa1265, aa1261-aa1269, and aa1265-aa1273 mutation sites, the overall affinity was more affected by the mutation, whereas the affinity before and after the aa1265-aa1273 mutation was still predominantly that of the standard strain IbAr1020.

### 3.8 ELISpot test validation

**Figure 4:**
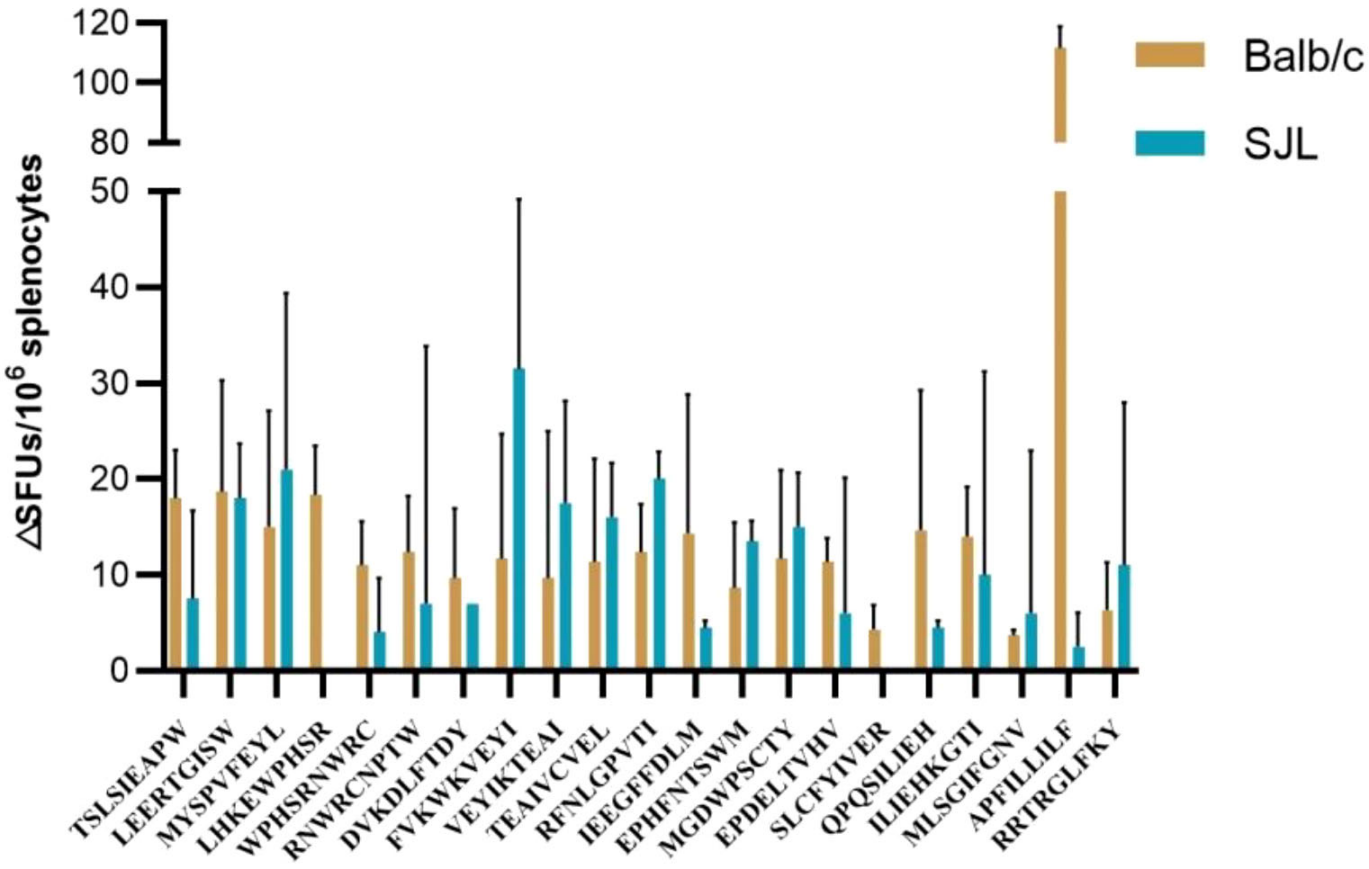
ELISpot assays of 21 high affinity, strong immunogenicity, and intra- & inter-species conservated epitope. IL-2 ELISpot response targeting predicted CCHFV epitopes. Data represent the mean of n=3 biological replicates (single mice) per group. Each biological replicate underwent triplicate technical replicates. The results are shown as the average of spot-forming units (SFUs) per 10^6^ splenocytes. The ordinate represents the difference between the experimental and the control groups (SUFs/10^6^ splenocytes) and the abscissa represents the different peptides for stimulation.

In the preliminary screening, 21 epitopes exhibiting high affinity, strong immunogenicity, and intra- & inter-species conservated were selected and artificially synthesized for validation by ELISpot assay. After immunization of BALB/c and SJL mice twice with pVAX-Gc CCHF plasmid, the splenocytes of BALB/c and SJL mice were stimulated with peptides to determine the ability of Th1-type cells to detect antigen-specific T cells by observing the secretion level of IL-2. Values are mean splenocyte spot forming units (SFU) (37). As shown in **Figure 4**, all 21 epitopes which stimulated splenocytes from BALB/c mice were able to secrete IL-2. 19 epitopes in SJL mice also showed splenocyte responses, indicating a good immune effect. With the notable epitope peptide, APFILLILF, inducing the strongest immune responses and release of the cytokine IL-2 from splenocytes in BALB/c mice, and the significant epitope peptide, FVKWKVEYI, inducing the strongest immune responses and release of the cytokine IL-2 from splenocytes in SJL. In SJL mice, the FVKWKVEYI epitope also responded significantly more strongly than the other epitopes. Other dominant epitopes such as LEERTGISW, MYSPVFEYL, and RFNLGPVTI also excelled in stimulating splenocytes and releasing cytokine IL-2 in both types of mice.

## 4 Discussion

In this study, the cellular immune response properties of CCHFV Gc 9-mer peptides were investigated using informative immunology and animal experiments. The affinity of the full-length CCHFV Gc 9-mer peptides for MHC I was determined in a pan-MHC context using predictive modeling and algorithms. The results identified 94 high-affinity epitopes on human HLA- I haplotypes and 37 high-affinity epitopes on mouse H2 haplotypes. Our validation of these MHC-I epitopes derived from the Gc protein was directly based on a CCHFV whole-protein DNA vaccine constructed previously (19) using a LAMP1 fusion Gc protein. This demonstrated that while the Gc vaccine could induce specific cellular and humoral immunity, its protective efficacy remained limited. This study began by deciphering key immunogenic epitopes mediating T-cell recognition, incorporating immunogenicity into the evaluation framework, and ultimately identified 109 highly immunogenic epitopes. To assess the conservation of the 9-mer peptides, we performed a conservation analysis of all peptides, resulting in the identification of 35 conserved epitopes across and within species. Combining these epitope characteristics, we identified 21 epitopes with high affinity, immunogenicity, and inter- and intra-specific conservation. By predicting population coverage, we clarified that the global population coverage could reach 98.55%. For safety evaluation, comprehensive toxicity and sensitization assessments demonstrated that the vast majority of screened epitopes exhibit favorable safety profiles. The 21 screened epitopes were tested by molecular docking simulation and mouse experiments, which demonstrated that these epitopes are effective in recognizing and generating specific T-cell immune responses from various perspectives.

To screen for high-affinity CTL epitopes, we utilized four algorithms to simulate the immune response activation process (38, 39). This resulted in a homogeneous data set to assess the binding affinities between the 9-complex epitopes and MHC-I molecules. The dataset was then used to evaluate the affinity between 9-mer epitopes and MHC-I, ensuring optimal activation of CTL cells and 9-mer epitopes. The research methodology used in this study avoids relying on a single tool to determine relevant affinities, resulting in more rigorous results. The findings indicate that the HLA-A2, HLA-A3, and HLA-B7 superfamilies ranked among the top three affinities for the selected CTL epitopes. It is worth noting that these three superfamilies cover nearly 90% of the global population, regardless of ethnicity. The screening process identified high-affinity epitopes in multiple HLA alleles across the three superfamilies, addressing the challenge posed by HLA polymorphism and population coverage (40).

Affinity is not the sole determinant of the immune response. The response is elicited when an antigen is both immunoreactive and immunogenic. Immunogenicity often refers to the ability of an MHC-presented peptide to activate an immune response (41). Therefore, in epitope studies, it is common practice to analyze the immunogenicity of epitopes using computational tools. The choice of computational tools is a critical part of the epitope immunogenicity study process. In this study, we evaluated and analyzed all Gc 9-mer peptide epitopes for immunogenicity using two different computational tools, PAAQD2.0 and IEDB. This approach helped us exclude high-affinity non-immunogenic epitopes and reduce the bias of a single computational tool.

Since its discovery in the 1960s, CCHFV has undergone independent evolution during transmission, resulting in multiple regional outbreaks and widespread epidemics. The distribution of tick-borne disease vectors has also been expanding, increasing the probability of geographic isolation and independent evolution of virulent strains. The genetic diversity of CCHFV resulting from these factors may lead to viral escape. For instance, in experiments where mice were challenged with heterologous CCHFV strains, incomplete protection was observed. Therefore, selecting conserved regions on standard strains as targets is crucial for subsequent immunization studies and development (42). We conducted interspecies and intraspecies conservation analyses of pre-existing high-affinity and strongly immunogenic epitopes, which we grouped into four categories based on interspecies and intraspecies conservation scores. In our results, HLA-B7 recognized the highest number of conserved epitopes. This suggests that the HLA-B7 epitope superfamily may elicit better immune responses in different strains during epidemics, thus providing better cross-protection based on cellular immune responses.

The overall affinity trend of CCHFV Gc 9-mer peptides with MHC is unknown, and the relationship between MHC affinities has not been elaborated. We performed a hierarchical cluster analysis of the MHC affinity of CCHFV Gc 9-mer peptides with 36 human and mouse genotypes representing different regions. There were two main findings, the first being that the proximity of most HLA I genotype clusters tends to imply that broader population protection is available. In our clustering results, the clusters of HLA-A32:01, HLA-B57:01, and HLA-B58:01 with the CCHFV Gc 9-mer peptide showed similar trends in the binding hotspot map. The HLA-B7 and HLA-A1 superfamilies showed similar results, which led us to move from the perspective of a single affinity analysis to that of the entire HLA, further elucidating the HLA cross immunoprotected capacity between CCHFV Gc 9 peptides. On the other hand, we found that some members of the human HLA superfamily and the mouse H2 superfamily showed a high degree of similarity in presenting the same Gc 9-mer peptide. Not only was it observed that the H-2Kd haplotype gene showed a similar clustering trend to the HLA-A*23:01 and HLA-A*24:01 alleles, but it was also found that the H-2Ld haplotype gene was located in the same region close to the HLA-B*08:01 and HLA-B*07:02 alleles. This overall cross-species affinity similarity between humans and mice suggests that BALB/c mice with MHC haplotypes H-2Kd and H-2Ld can express some of the response in place of humans in experimental models where humanized HLA-I transgenes are not available, and provide a reasonable animal model reference for subsequent relevant immunological studies.

Previous studies have convincingly demonstrated that CCHFV, a tick-borne hemorrhagic fever virus with a vast geographic distribution, ranks among the most genetically diverse arboviruses. Its segmented genome facilitates recombination events, thereby expediting the emergence of infectious strains capable of causing outbreaks (43). The introduction of mutant strains into circulation introduces novel uncertainties regarding the efficacy of current vaccines and escalates the risk of infection among high-risk populations (44). Globally, while NP and L proteins of diverse CCHFV strains exhibit remarkable conservation (45), with amino acid conservation rates exceeding 95% among strains, the GP displays less promising conservation levels, with only 75% amino acid conservation across strains (46).Consequently, investigating the variation in CCHFV antigens is imperative Interestingly, the hydrolysis of GPC to produce Gn and Gc has been reported to be restrictively protected during processing and transport (44), but it remains to be determined whether our chosen Gc epitope is also highly conserved in the strain. After analyzing the epitopes with high mutation rates, it was found that only 4 of the 10 preferred sites showed a tendency to bind MHC-I molecules from different strains. This observation confirms the intraspecific conservation of these epitopes in Gc and also indicates that these epitopes are located in highly conserved regions of Gc. The study and development of these epitopes would be an effective approach to counter the highly genetically diverse immune escape of CCHFV.

Whilst computational prediction efficiently identifies high-affinity epitopes, the dynamic nature of peptide-MHC interactions necessitates structural validation for a comprehensive understanding of their immunogenic potential. Molecular docking and molecular dynamics simulations represent complementary yet distinct structural biology tools. This study prioritises molecular docking (via HPEPDOCK 2.0) for high-throughput prediction to determine epitope-MHC-I binding conformations, owing to its effective balance between computational efficiency and predictive capability. Concurrently, we observed that given the HLA-B7-restricted CCHFV Gc epitope exhibits pan-MHC-I characteristics, cluster analysis indicates that HLA-B*0702 alleles within the HLA-B7 superfamily demonstrate antigen-presenting tendencies similar to H2-Ld.We verified by ELISpot experiments that all these epitopes elicited a certain amount of antibodies in BALB/c mice, which further confirmed our immunological analysis of these epitopes. Meanwhile, Gabrielle Scher et al. have shown that the Th1-related response induced by the CCHFV vaccine is quite important for protection (2, 14, 47). Furthermore, the correlation between epitope conservation, mutant affinity stability, and ELISpot responses provides additional validation for the reliability of our epitope screening approach. In viral immunology, conserved epitopes are vital for cross-strain protection as they circumvent immune evasion triggered by viral mutations (19). Among the screened epitopes, the highly conserved epitope APFILLILF induced immune responses over three times stronger than other epitopes, demonstrating its capacity to elicit robust anti-CCHFV-specific cellular immune responses in BALB/c mice. This further underscores the necessity and value of developing and investigating these antigenic epitopes.

Computer-assisted immuno-design of epitopes has become a hotspot and frontier in the study of CCHFV. However, the limitations of computer-aided immuno-design must not be overlooked. For instance, in epitope conservation analysis, conventional methods rely solely on the BLASTp conservation prediction tool, and the results fail to demonstrate the correlation between epitope conservation, mutation affinity stability, and ELISpot responses. Despite the singularity of the traditional idea, we still propose a comprehensive analysis concept for highly mutated loci, which not only further tests the results of the traditional conservation prediction and reduces the chance error caused by a single prediction tool, but also focuses on highly mutated loci by using tools such as WebLogo and reduces the repetitive work of experiments and the waste of time. The accuracy of this level of validation of conservation is further improved by further analysis of the highly mutated loci of our screened epitopes. Our epitope screening idea also analyzed and validated affinity in multiple dimensions, laying the foundation for subsequent epitope research and vaccine development from multiple perspectives. Additionally, the epitope’s antigenicity, sensitization, virulence and population coverage can be used as a reference for the design of other viral epitopes and vaccines.

## 5 Conclusion

In this study, we established the immunological evaluation of pan-MHC-I epitopes on CCHFV Gc through a comprehensive combination of computational 9-peptide epitope assessment and animal experiments. Through integrated analysis of affinity, immunogenicity, toxicity, sensitization potential, and conservation, we identified 21 superior epitopes exhibiting high affinity for human HLA-I and murine H2 alleles, strong immunogenicity, and conservation across and within species. Several epitopes induced robust epitope-specific T-cell responses in mice, confirming their biological relevance. These Gc epitopes provide an ideal reference for designing multivalent vaccines and elucidating the T-cell immune mechanisms against CCHFV. Subsequent work will focus on structurally characterizing major epitope-MHC complexes, extending validation to additional HLA backgrounds and non-inbred mouse models, and exploring the integration of these epitopes with suitable adjuvants to create optimized multivalent immunogens. Their protective efficacy against CCHFV will be systematically evaluated.

## Data Availability

The original contributions presented in the study are included in the article/**Supplementary Material**. Further inquiries can be directed to the corresponding author.
