# Supplementary material for: Immunoreactivity of pan MHC-I epitopes on Crimean-Congo hemorrhagic fever virus glycoprotein C-terminal
Source: Front Immunol. 2026 Mar 4;17:1693892. doi: 10.3389/fimmu.2026.1693892 (PMC12996153; doi:10.3389/fimmu.2026.1693892)
Supplement: Supplementary file 1 [file DataSheet1.pdf]

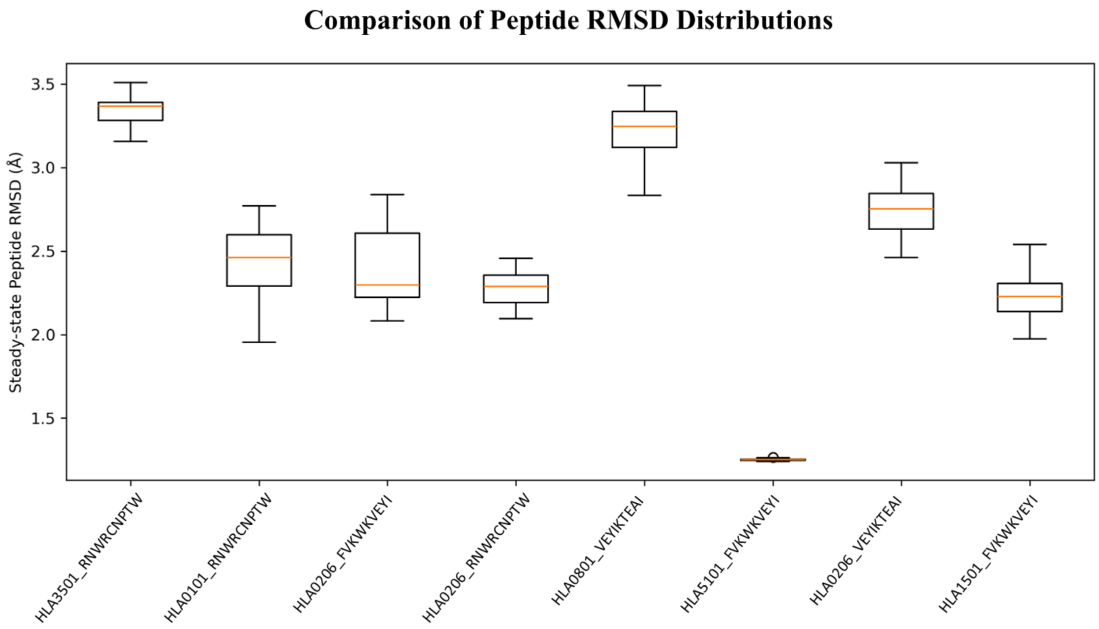

Supplementary Figure S1. Comparison of peptide RMSD distributions among simulated pMHC complexes. Box plots show the steady-state distributions of peptide heavy-atom RMSD (Å) for each HLA–peptide pair, illustrating differences in peptide conformational stability within the binding groove and highlighting the particularly rigid FVKWKVEYI peptide in complex with HLA-B*51:01.

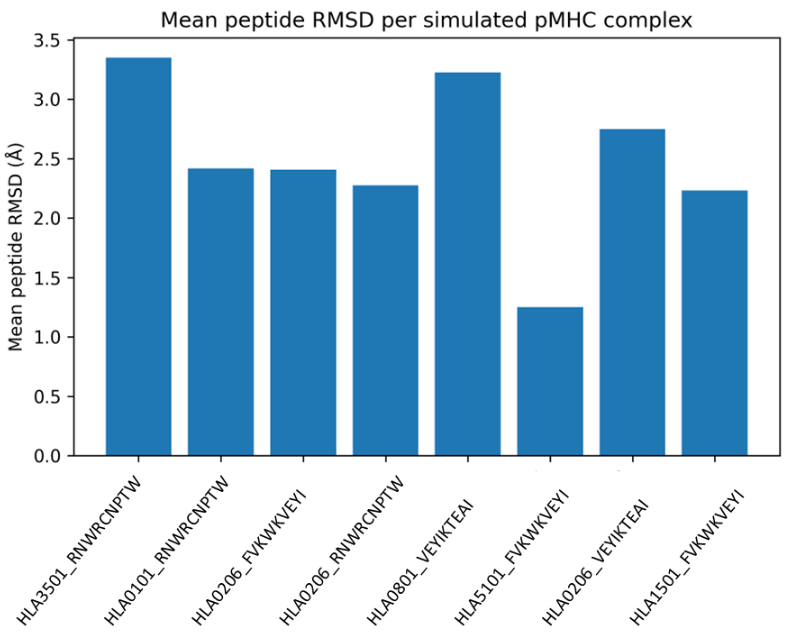

Supplementary Figure S2. Mean peptide RMSD across simulated pMHC complexes. Bars represent the mean steady-state peptide RMSD (Å) for each HLA–peptide combination, summarizing overall peptide flexibility and indicating which complexes maintain more constrained peptide conformations that are compatible with stable antigen presentation.

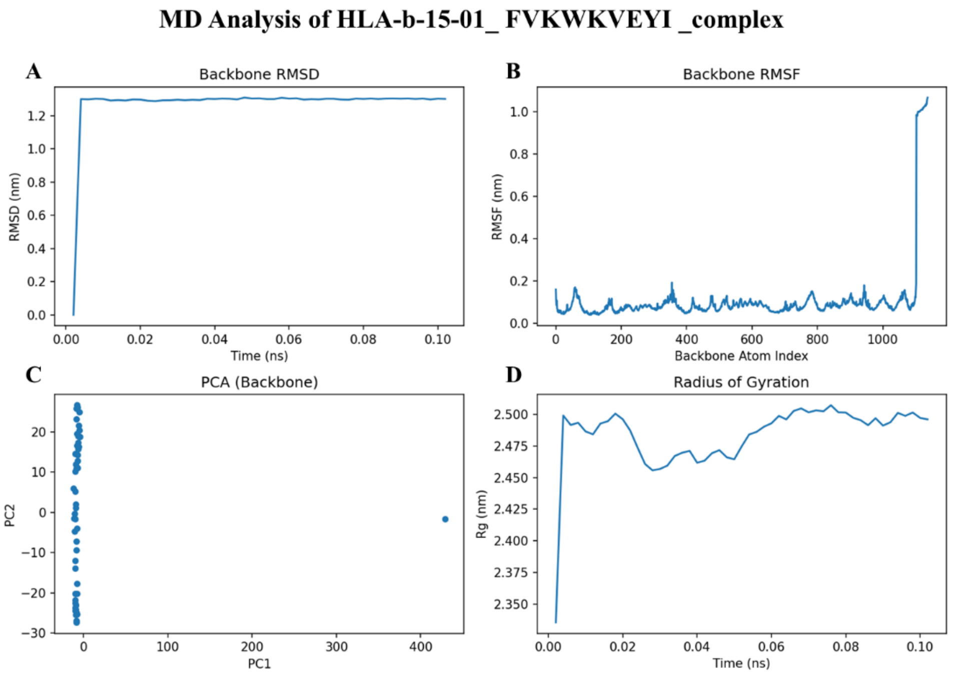

Supplementary Figure S3. MD analysis of the HLA-B*15:01–FVKWKVEYI complex. (A) Backbone RMSD as a function of time, showing rapid equilibration and a stable global conformation over the simulation.(B) Backbone RMSF per atom index, indicating limited flexibility in the peptide-binding groove and increased motion only at terminal regions.(C) Principal component analysis (PCA) of backbone motions, demonstrating that the complex samples a relatively restricted conformational space.(D) Radius of gyration over time, confirming that the pMHC complex remains compact, consistent with stable binding of FVKWKVEYI by HLA-B*15:01.

MD Analysis of HLA-a-02-06_ VEYIKTEAI _complex

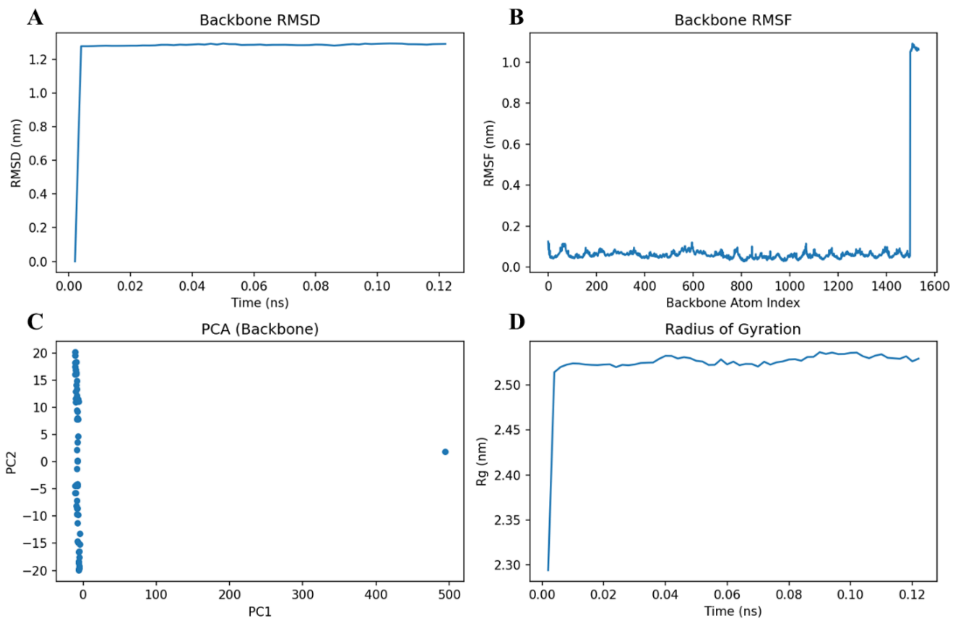

Supplementary Figure S4. MD analysis of the HLA-A*02:06–VEYIKTEAI complex. (A) Backbone RMSD over time, indicating rapid convergence to a stable conformation.(B) Backbone RMSF profile, showing low fluctuations in the HLA binding groove with higher mobility confined to terminal regions.(C) PCA of backbone dynamics, suggesting that the complex explores a narrow ensemble of conformations.(D) Time evolution of the radius of gyration, demonstrating a stable and compact pMHC architecture compatible with robust epitope presentation.

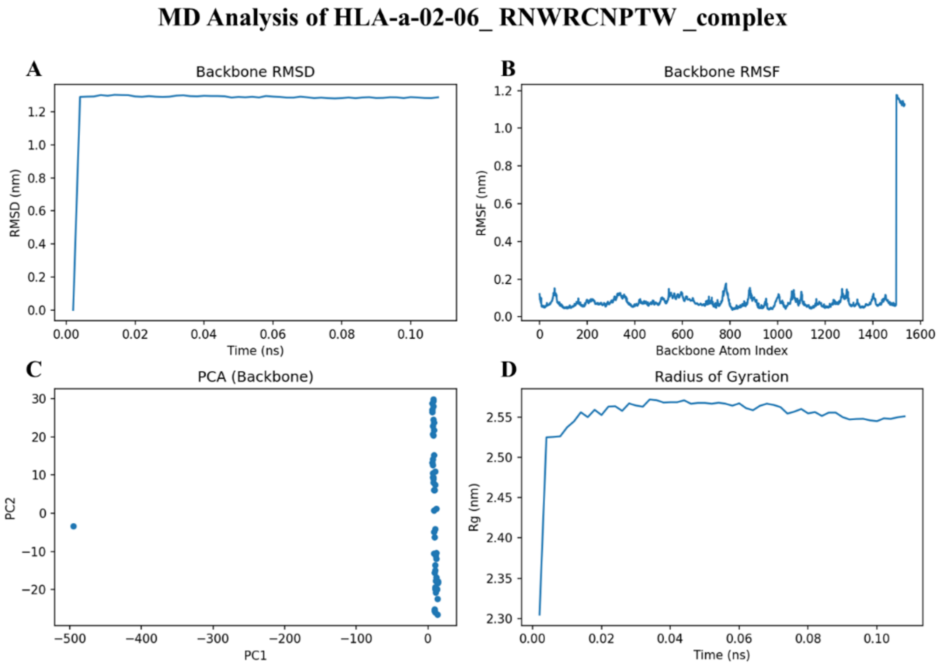

Supplementary Figure S5. MD analysis of the HLA-A*02:06–RNWRCPNTW complex. (A) Backbone RMSD as a function of time, showing fast equilibration and stable backbone geometry.(B) Backbone RMSF per atom index, revealing limited local flexibility in the peptide-binding groove and modest fluctuations in peripheral regions.(C) PCA scatter plot of backbone motions, indicating that the complex predominantly occupies a single conformational basin.(D) Radius of gyration over time, supporting a compact and structurally stable pMHC complex for RNWRCPNTW bound to HLA-A*02:06.

MD Analysis of HLA-a-02-06_ FVKWKVEYI _complex

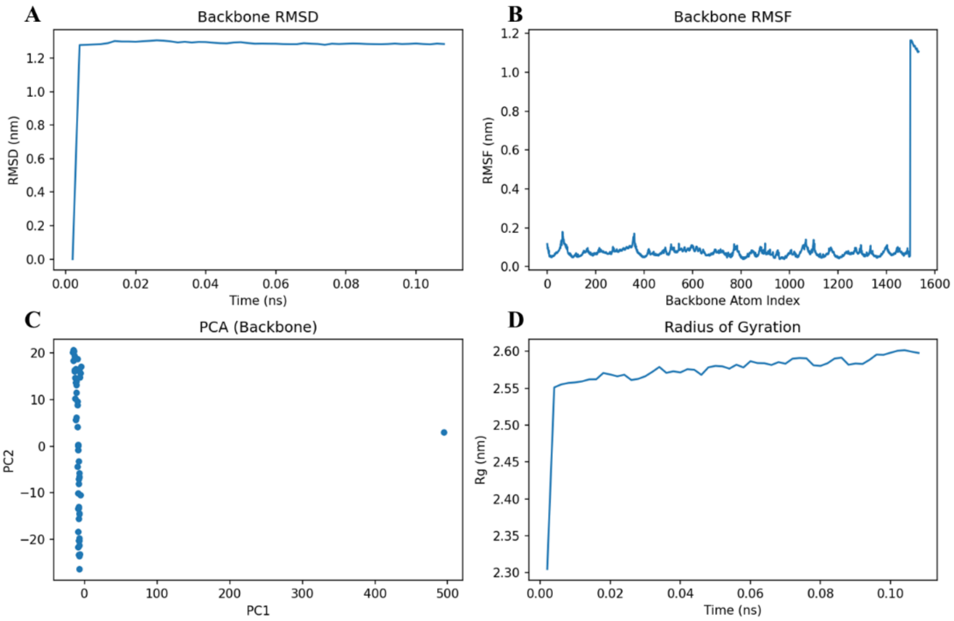

Supplementary Figure S6. MD analysis of the HLA-A*02:06–FVKWKVEYI complex. (A) Backbone RMSD trace, demonstrating early stabilization of the complex during the simulation.(B) Backbone RMSF along the sequence, showing low flexibility in the peptide-binding site and enhanced motion only at the termini.(C) PCA of backbone motions, with most frames clustering tightly, consistent with restricted conformational sampling.(D) Radius of gyration versus time, indicating that the HLA-A*02:06–FVKWKVEYI complex maintains a compact fold compatible with stable peptide presentation.

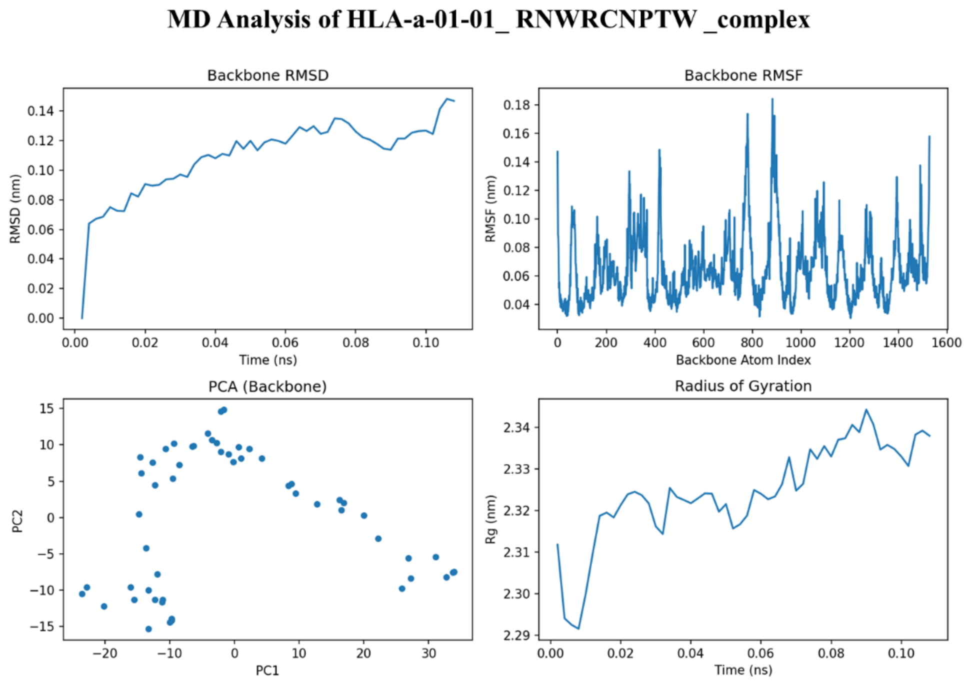

Supplementary Figure S7. MD analysis of the HLA-A*01:01–RNWRCPNTW complex. (A) Backbone RMSD over time, showing a gradual increase that reflects more pronounced backbone rearrangements.(B) Backbone RMSF profile, revealing elevated flexibility across multiple backbone segments compared with other complexes.(C) PCA scatter plot of backbone dynamics, indicating broader conformational sampling and a more heterogeneous ensemble.(D) Radius of gyration as a function of time, showing modest expansion of the complex, consistent with increased structural plasticity of this pMHC.

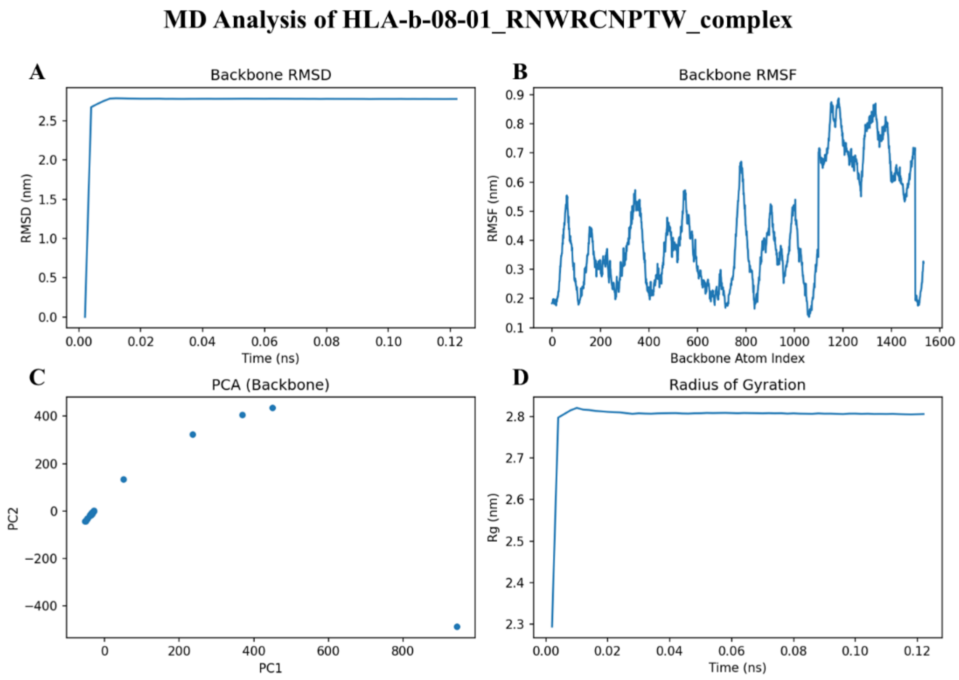

Supplementary Figure S8. MD analysis of the HLA-B*08:01–RNWRCPNTW complex. (A) Backbone RMSD trace, indicating rapid equilibration to a higher-RMSD but stable conformation.(B) Backbone RMSF along the sequence, highlighting pronounced flexibility in several backbone regions, including parts of the binding groove.(C) PCA of backbone motions, demonstrating that the complex explores a wide conformational space with multiple dominant modes.(D) Radius of gyration over time, showing a relatively expanded yet stable global architecture of the HLA-B*08:01–RNWRCPNTW complex.

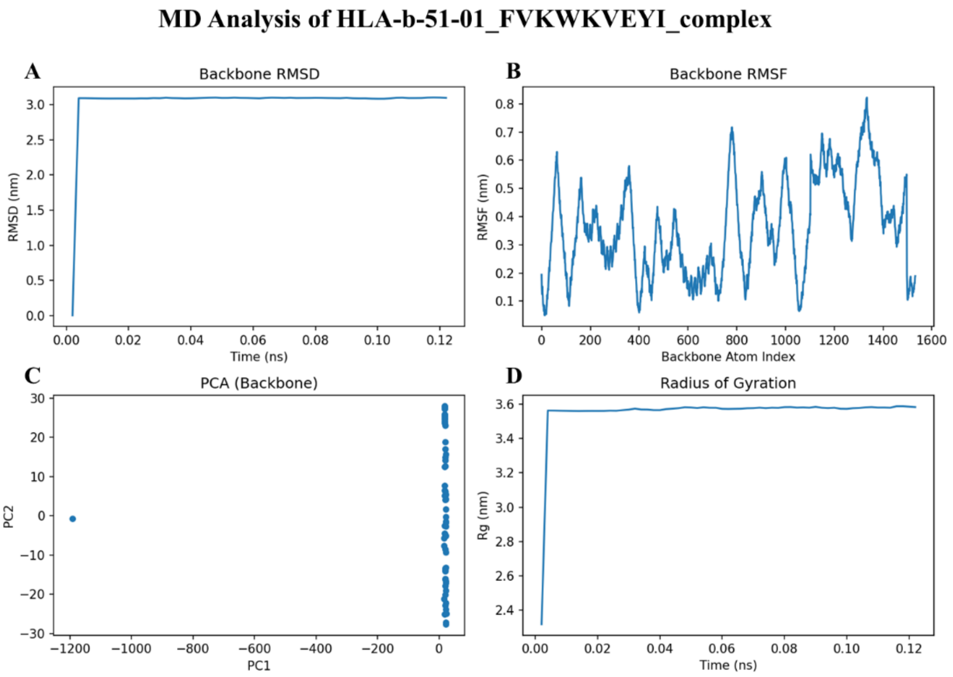

Supplementary Figure S9. MD analysis of the HLA-B*51:01–FVKWKVEYI complex.(A) Backbone RMSD as a function of time, showing rapid convergence and exceptionally stable backbone geometry.(B) Backbone RMSF profile, indicating substantial flexibility in loop and terminal regions but preserved rigidity in the peptide-binding groove.(C) PCA scatter plot of backbone dynamics, in which most conformations cluster tightly, consistent with a highly constrained ensemble.(D) Radius of gyration over time, confirming a compact and stable pMHC framework that supports tight presentation of FVKWKVEYI by HLA-B*51:01.

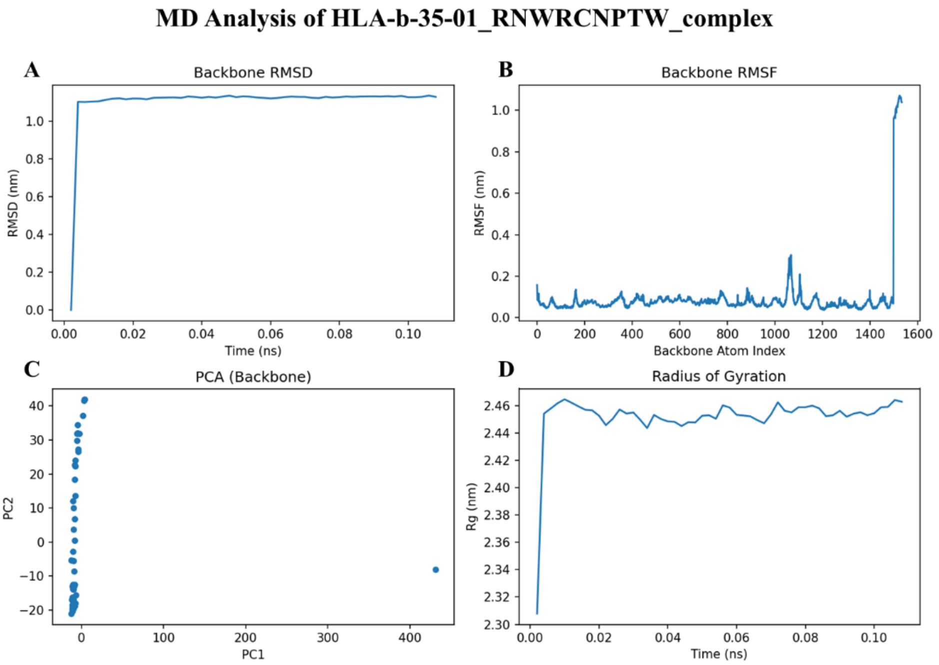

Supplementary Figure S10. MD analysis of the HLA-B*35:01–RNWRCPNTW complex.(A) Backbone RMSD trace, demonstrating rapid equilibration and sustained structural stability.(B) Backbone RMSF per atom index, showing generally low fluctuations with localized flexibility at the C-terminal region.(C) PCA of backbone motions, indicating that most conformations reside within a single major cluster.(D) Radius of gyration versus time, supporting a compact and stable HLA-B*35:01–RNWRCPNTW pMHC complex compatible with efficient epitope presentation.
